# Serine acetyltransferase from *Neisseria gonorrhoeae*; structural and biochemical basis of inhibition

**DOI:** 10.1042/BCJ20210564

**Published:** 2022-01-06

**Authors:** Keely E. A. Oldham, Erica J. Prentice, Emma L. Summers, Joanna L. Hicks

**Affiliations:** 1 Te Aka Mātuatua School of Science, University of Waikato, Hamilton, New Zealand; 2 Te Huataki Waiora School of Health, University of Waikato, Hamilton, New Zealand

**Keywords:** CysE, cysteine, cysteine biosynthesis, gonorrhoea, *Neisseria gonorrhoeae*, serine acetyltransferase

## Abstract

Serine acetyltransferase (SAT) catalyzes the first step in the two-step pathway to synthesize l-cysteine in bacteria and plants. SAT synthesizes *O*-acetylserine from substrates l-serine and acetyl coenzyme A and is a key enzyme for regulating cellular cysteine levels by feedback inhibition of l-cysteine, and its involvement in the cysteine synthase complex. We have performed extensive structural and kinetic characterization of the SAT enzyme from the antibiotic-resistant pathogen *Neisseria gonorrhoeae*. Using X-ray crystallography, we have solved the structures of NgSAT with the non-natural ligand, l-malate (present in the crystallization screen) to 2.01 Å and with the natural substrate l-serine (2.80 Å) bound. Both structures are hexamers, with each monomer displaying the characteristic left-handed parallel β-helix domain of the acyltransferase superfamily of enzymes. Each structure displays both extended and closed conformations of the C-terminal tail. l-malate bound in the active site results in an interesting mix of open and closed active site conformations, exhibiting a structural change mimicking the conformation of cysteine (inhibitor) bound structures from other organisms. Kinetic characterization shows competitive inhibition of l-cysteine with substrates l-serine and acetyl coenzyme A. The SAT reaction represents a key point for the regulation of cysteine biosynthesis and controlling cellular sulfur due to feedback inhibition by l-cysteine and formation of the cysteine synthase complex. Data presented here provide the structural and mechanistic basis for inhibitor design and given this enzyme is not present in humans could be explored to combat the rise of extensively antimicrobial resistant *N. gonorrhoeae*.

## Introduction

*Neisseria gonorrhoeae* is an obligate human pathogen that causes the sexually transmitted infection gonorrhoea. With purported references to gonorrhoea in early texts including the bible [1] the bacterium *N. gonorrhoeae* has plagued humans for centuries. Of the many and varied treatments developed over decades, *N. gonorrhoeae* has developed resistance to nearly all of the antibiotics used for its treatment. As a consequence, we are now facing the reality of untreatable gonorrhoea with only one effective class of antibiotics, the cephalosporin's left to treat this common infection. With no vaccine available, gonorrhoea control relies on our ability to treat gonorrhoea infections and new antimicrobials are desperately needed for control of this disease in the future.

The failure to identify new targets for antibiotic treatment has been shown by the small number of antibiotics discovered in the last 50 years and can be attributed in part to a lack of knowledge of microbial metabolism inside the host [2]. Targeting amino acid biosynthesis is a new and promising route for the development of new antimicrobials [3,4]. Synthesis of the amino acid cysteine is the primary pathway for the incorporation of sulfur into a variety of important cellular constituents including methionine, thiamin, biotin and coenzyme A. Cysteine also plays important roles in protein molecules in Fe–S clusters, catalysis, protein folding and the formation of disulfide bonds. In microorganisms, cysteine provides protection from oxidative stress via reducing systems such as thioredoxin and glutathione.

In most bacteria, the cysteine biosynthesis pathway is well conserved. Sulfate is transported into the cell and via a series of enzymatic steps is successively reduced to sulfide via the sulfate reduction pathway, which is then incorporated into cysteine [5]. Under anaerobic conditions, thiosulfate is imported and *S*-sulfocysteine is produced which is then converted into cysteine by an uncharacterized process.

SAT (annotated as CysE) catalyzes the first step in the two-step reaction that makes l-cysteine from l-serine. The side chain hydroxyl of l-serine undergoes a coenzyme A-dependent acetylation to form *O*-acetylserine [6]. The second step is then catalyzed by one of two OASS isoforms; OASS-A/CysK which catalyzes the β-replacement of acetate with sulfide to convert *O*-acetylserine to l-cysteine [7], or OASS-B/CysM which catalyzes the β-replacement of acetate with thiosulfate to form S-sulfocysteine [8], which is converted to l-cysteine via an unknown mechanism.

Bacteria generally have both OASS-A and OASS-B isoforms, thereby capable of growth on sulfate or thiosulfate as the sole source of sulfur. Neisseria species appear to have only one OASS isoform annotated as CysK (OASS-A) [9], yet unlike other Neisseria species, *N. gonorrhoeae* cannot grow on sulfate as the sole source of sulfur [10] due to a 3.5 kb deletion in the sulfate reduction operon compared with other Neisseria species [9]. However, all Neisseria strains, including *N. gonorrhoeae*, are able to use thiosulfate as a sole source of sulfur [10], which suggests that functional SAT and OASS enzymes are present.

SAT and OASS form a bi-enzyme complex termed the cysteine synthase complex which is intricately involved in the control of sulfur metabolism [11,12]. Many bi-enzyme complexes channel substrates from one enzyme to another. However, the cysteine synthase complex is unusual in that the flexible C-terminal tail of SAT inserts into the active site of OASS competing with the binding of *O*-acetylserine and inhibiting OASS activity [13,14]. SAT activity is enhanced in the complex [12] and produces the pathway intermediate *O-*acetylserine which at high concentrations promotes dissociation of the complex [15] and subsequent consumption of *O-*acetylserine for cysteine synthesis. The OASS variant, OASS-B that utilizes thiosulfate to produce *S-*sulfocysteine does not form a complex with the SAT enzyme [16]. Due to differences in the pathways for sulfate reduction between other bacterial pathogens and *N. gonorrhoeae*, respectively, it is unknown if the SAT and OASS enzymes from *N. gonorrhoeae* form the cysteine synthase complex or how this regulates sulfur metabolism.

Other mechanisms of regulation for the *de novo* synthesis of cysteine include feedback inhibition of SAT by l-cysteine, transcriptional regulation of sulfur acquisition and cysteine biosynthetic genes by transcriptional regulator CysB regulating sulfur acquisition and changes in the quaternary structure of SAT [17,18]. Due to the unique nature of sulfur acquisition for cysteine biosynthesis in *N. gonorrhoeae*, the essential nature of SAT for growth in *N. gonorrhoeae* (NgSAT) [19] and the targeting of SAT in other pathogens [20–22], NgSAT could represent a novel drug target for the treatment of antimicrobial resistant gonorrhoea.

SAT, belongs to the family of *O*-acetyltransferases (EC 2.3.1.30) found in both plants and bacteria and there is good structural conservation between bacterial and plant SAT enzymes, even though there is a large degree of sequence divergence [23,24]. The *O*-acetyltransferase family is defined by the hexapeptide repeat sequence [LIV]-[GAED]-X_2_-[STAV]-X, that gives rise to the unique left-handed β-helix (LβH), characteristic of this family [25].

SAT enzymes consist of two structural domains, an N-terminal, α-helical domain and a C-terminal, LβH domain. The C-terminal LβH domain is structured like a triangular prism, which fits with two other monomers to form a trimer [26]. The resulting trimer dimerizes to form a hexamer through hydrophobic interactions at the N-terminal face of each trimer [6]. SAT enzymes are unique as they are the only hexapeptide acyltransferase to adopt a hexameric conformation, instead of the more common trimeric arrangement [26]. This higher oligomeric state allows for formation of the bi-enzyme cysteine synthase complex and regulation of sulfur flux [12,13]. To date the only instance of a trimeric SAT has been identified in the human parasite *Entamoeba histolytica* [27].

Members of the LβH family, including SAT have a conserved histidine in the active site [26]. The proposed mechanism for the acetyl-transfer reaction of SAT, is by general acid–base catalysis, where the histidine, alongside a conserved aspartate form a catalytic triad with the substrate l-serine [6,26]. In *Escherichia coli* this aspartate residue (Asp143) is positioned in close proximity (2.8 Å) to the well-conserved histidine (His158), which is predicted to transform the histidine into a strong base [6]. His158 activates the hydroxyl group of l-serine, for nucleophilic attack on the acetyl thioester of acetyl-CoA. The nucleophilic attack on acetyl-CoA forms a ternary complex between the enzyme, l-serine and acetyl-CoA, before the histidine behaves as a general acid and donates a hydrogen to the sulfur group of acetyl-CoA, leading to collapse of the complex and product release [6,26].

Initial research hypothesized a ping-pong reaction mechanism for the SAT [28]. However, a combination of equilibrium isotope exchange experiments [29] and Lineweaver–Burke plots [12,29,30] overwhelmingly supports a sequential binding mechanism. The exact type of sequential mechanism reported for SAT varies, with a random sequential mechanism reported for SAT from *E. coli* [29] and an ordered binding mechanism reported for SAT from *Haemophilus influenzae* [30], where acetyl-CoA binds first, followed by l-serine.

The cysteine biosynthetic pathway is feedback inhibited by l-cysteine binding to SAT. l-cysteine competes directly with l-serine for the active site displaying competitive inhibition [30]. Analysis of co-crystallized structures of SAT with l-cysteine, shows l-cysteine bound in the l-serine binding pocket located in the SAT active site [6,23,31], supporting the observation of l-cysteine being a competitive inhibitor relative to l-serine [5,30]. This mechanism of inhibition appears to be well-conserved across the SAT family [30,32]. Interestingly, l-cysteine has also been reported to be a competitive inhibitor relative to acetyl-CoA [30]. SAT structures with l-cysteine bound show conformational differences in the C-terminal tail compared with *apo*-SAT structures with the C-terminal tail folding against SAT to obscure the active site in l-cysteine-bound structures. This folded tail precludes acetyl-CoA from binding to the acetyl-CoA binding site [24] and prevents the unwanted acetylation of l-cysteine, given that it is isostructural to l-serine.

To design new antimicrobial inhibitors to target critical pathways such as cysteine biosynthesis in *N. gonorrhoeae* we need high quality, accurate three-dimensional structures of the target enzymes and a detailed understanding of enzymatic mechanism. To this end, we have determined the kinetic parameters and regulation of SAT from *N. gonorrhoeae* and the structure of this enzyme to 2.01 Å with the non-natural ligand, l-malate (from the crystallization condition) bound in the active site and with the natural substrate l-serine bound to 2.8 Å.

## Materials and methods

### Cloning of NgSAT for expression in *Escherichia coli*

The SAT gene (annotated *cysE*) NGFG_RS07905 (old locus tag NGFG_01496) from *N. gonorrhoeae* MS11 was codon optimized for *E. coli* and ordered from Geneart (Thermo Fisher). The synthetic NGFG_RS07905 construct was cloned into expression vector pET28b between Ndel and Xhol restriction sites for expression with an N-terminal His-tag. Insertion of NGFG_RS07905 into pET28b was confirmed by DNA sequencing prior to transformation into *E. coli* BL21 (DE3) for protein expression. Positive transformants were selected for by plating on Luria–Bertani (LB) agar supplemented with 50 μg.ml^−1^ kanamycin and incubating overnight at 37°C.

### NgSAT expression and purification

*E. coli* BL21 (DE3) containing the NGFG_RS07905_pET28b plasmid were cultured in 1 l LB broth, supplemented with 50 μg.ml^−1^ kanamycin. Cultures were incubated at 37°C (200 rpm) until OD_600_ reached between 0.5 and 0.7. Protein expression was induced by the addition of 0.75 mM IPTG and the cultures were incubated at 37°C (200 rpm) overnight. Cultures were centrifuged at 4600 ***g*** for 20 min at 4°C and the resulting cell pellet resuspended in lysis buffer (50 mM Tris pH 8.0, 200 mM NaCl, 20 mM imidazole). One Complete Mini, EDTA-free protease inhibitor tablet (Roche) was added prior to cell lysis by sonication. Lysate was centrifuged at 20 000 ***g*** for 20 min at room temperature and 0.2 μm filtered supernatant was loaded onto a pre-equilibrated HisTrap^TM^ column (GE Healthcare). The column was washed with 20 ml of lysis buffer before the elution of NgSAT using a 50% gradient over 25 ml (50 mM Tris pH 8.0, 200 mM NaCl, 1 M imidazole).

Immobilized-metal ion affinity chromatography-purified NgSAT was concentrated at 15°C using a spin concentrator to a final volume of 0.75 ml. Concentrated NgSAT was loaded and run through an Enrich 650 analytical gel filtration column (Bio-Rad), pre-equilibrated in 50 mM Tris pH 8.0, 100 mM NaCl and eluted NgSAT was collected and stored at room temperature. Protein concentration was measured by absorbance at 280 nm by Nanodrop^TM^.

### Measuring the oligomeric state of NgSAT

An Enrich 650 gel filtration column (Bio-Rad) was calibrated in 50 mM Tris pH 8.0, 100 mM NaCl using Gel Filtration Standards (Bio-Rad) according to manufacturer's instructions.

### NgSAT kinetic assays

NgSAT for kinetic characterization was purified immediately prior to assays. Enzyme was stored at room temperature for the duration of the assay, as a rapid decrease in activity was observed when stored on ice. Assays were conducted within 2 h post-purification as NgSAT activity started to decrease after 2 h at room temperature (Supplementary Figure S1). NgSAT activity was measured by adapting the method in [33]. NgSAT activity was measured by monitoring the decrease in absorbance at 232 nm (*A*_232_) due to breakage of the thioester bond of acetyl-CoA (Δ*ε*232 = 4500 M^−1 ^cm^−1^) using a Thermo Spectronic Heλios spectrophotometer (Thermo Fisher).

To calculate the *K*_M_ and *k*_cat_ for the substrate l-serine, assays were carried out in quartz cuvettes of 0.5 cm pathlength with a final reaction volume of 0.4 ml which contained 50 mM Tris pH 8.0, 100 mM NaCl, 0.45 mM acetyl-CoA and variable amounts of l-serine. The reaction was performed at 22°C with absorbance recorded every 0.125 s after the reaction was initiated by the addition of 0.625 µg of purified NgSAT. Enzyme concentration in activity assays was optimized by testing various concentrations of NgSAT (0.156, 0.312, 0.391, 0.521, 0.781 and 1.56 µg.ml^−1^; Supplementary Figure S2). All substrate stocks were prepared in 50 mM Tris pH 8.0, 100 mM NaCl. An enzyme working stock of 0.125 mg.ml^−1^ (3.96 µM, NgSAT monomer 31.6 kDa) was stored at room temperature (22°C) for the duration of the assays. The *K*_M_ and *k*_cat_ was calculated for acetyl-CoA, by varying the amount of acetyl-CoA and keeping the concentration of l-serine constant at 10 mM. *K*_M_ and *k*_cat_ values were determined by non-linear regression fit of the Michaelis–Menten equation (eqn 1) using GraphPad Prism (GraphPad Software Version 8.2.0). The initial velocity of the reaction was derived from linear-regression analysis of the first five seconds of the reaction. All concentrations were collected in duplicate. Rates were divided by enzyme concentration before plotting substrate concentration versus rate. Substrate inhibition was modeled for acetyl-CoA through non-linear regression fit of the substrate inhibition equation (eqn 2) using GraphPad Prism (GraphPad Software Version 8.2.0).

Michaelis–Menten equation:

1 $$\text{Rate}=\;\frac{V_{\max}\;[S]}{K_{M}+[S]}\;$$

Substrate inhibition equation:

2 $$\text{Rate}=\;V_{\max}\frac{\left[S\right]}{K_{M}+[S]\left(1+\frac{\left[S\right]}{K_{i}}\right)}$$

### NgSAT inhibition assays

To calculate the IC_50_ for l-cysteine, similar reactions to above were set up with the addition of l-cysteine (final concentration, 0.01–40 μM) to the assay reaction before initiation of the reaction by the addition of NgSAT. l-cysteine stocks (0.1 and 1.0 mM) were made fresh in 50 mM Tris pH 8.0, 100 mM NaCl, and stored on ice to prevent unwanted oxidation of l-cysteine to l-cystine. The IC_50_ for coenzyme A (CoA) was calculated in a similar manner to cysteine, which involved the addition of CoA (final concentration, 10–2000 μM), before initiating the reaction with NgSAT. The IC_50_ values for l-cysteine and CoA were determined by fitting eqn 3 using GraphPad Prism (GraphPad Software Version 8.2.0) [34,35].

Log (inhibitor) versus normalized response — variable slope equation:

3 $$\text{Rate}=\frac{100}{\left(1+10^{\left((\mathrm{LogI}C_{50}-X)\mathrm{HillSlope}\right)}\right)}$$

Michaelis–Menten plots for substrates acetyl-CoA and l-serine were collected as above with the addition of 4, 6 and 8 µM l-cysteine, respectively, to determine the mode of inhibition relative to each substrate. For varying concentrations of l-serine, acetyl-CoA was held constant at 0.15 mM and for varying concentrations of acetyl-CoA, l-serine was held constant at 1.5 mM. Inhibition data were analyzed using the mixed-model inhibition equation (eqn 4) in GraphPad Prism (GraphPad Software Version 8.2.0) to determine the mode of inhibition for each substrate. *K*_M_ and *k*_cat_ values for each cysteine concentration relative to each substrate were determine by non-linear regression of the Michaelis–Menten equation (eqn 1) in GraphPad Prism (GraphPad Software Version 8.2.0).

Mixed-model inhibition equation:

4 $$\text{Rate}=V_{\max}\frac{\left[S\right]}{[S]\left(1+\frac{\left[I\right]}{\alpha K_{i}}\right)+K_{M}\left(1+\frac{\left[I\right]}{K_{i}}\right)}$$

### Crystallization of NgSAT

Initial crystals were grown by vapor diffusion using an Index^TM^-HR2-144 crystallization screen (Hampton Research), dispensed into a low profile 96-2 well Intelli Plate (Hampton Research) using a Mosquito crystallization robot (TTP LabTech Ltd). Sitting drops consisted of a 1 : 1 mix (100 nl :100 nl) of reservoir solution and concentrated protein (35 mg.ml^−1^ of purified NgSAT) with 100 μl in the reservoir well. Crystals for diffraction collection were grown using hanging drop vapor diffusion at 18°C. NgSAT crystals were obtained using whisker seeding in 28% (*v/v*) Tacsimate^TM^ pH 7.0 (0.3 M DL-malic acid, 1.8305 M malonic acid, 0.25 M ammonium citrate tribasic, 0.12 M succinic acid, 0.4 M sodium acetate trihydrate, 0.5 M sodium formate and 0.16 M ammonium tartrate dibasic, Hampton Research). Drop composition consisted of a 1 : 1 mix (2 µl : 2 µl) of reservoir and protein with 500 µl in the reservoir well. NgSAT with serine bound (NgSAT + l-Ser) crystals were obtained using similar techniques, but were grown using 100 mM Tris pH 8.4, 26% (*v*/*v*) PEG400 and 15 mM serine (24 mg.ml^−1^ of purified NgSAT). For data collection, all crystals were transferred to a cryo-protectant solution, consisting of crystallization solution with 15% (*v/v*) glycerol, prior to flash cooling in liquid nitrogen.

### Data collection, indexing, integration and scaling

X-ray diffraction data (100 K) was collected at the Australian Synchrotron at the MX2 beamline [36] equipped with an EIGER ×16M detector (Dectris).

The NgSAT and NgSAT + l-Ser dataset diffraction images were indexed, integrated and scaled, using XDS [37]. Merging of reflections was carried out using AIMLESS [38] from the CCP4 suite [39]. Data quality was assessed through AIMLESS [38] and through manually viewing diffraction images using ALBULA (Dectris). The total number of monomers in the asymmetric unit was determined by calculating the solvent content using the Matthew's coefficient program [40], as a part of the CCP4 program suite [39]. The R free flag dataset was generated in AIMLESS [38].

Data were analysed for evidence of twinning and translational non-crystallographic symmetry (tNCS) in AIMLESS from the CCP4 suite [38,39] and *phenix.xtriage* from the Phenix suite [41].

### Structure building and refinement

The NgSAT structure was solved using molecular replacement, using the structure of SAT from *Yersinia pestis* (3GVD) retrieved from the protein data bank (PDB). A single monomer was extracted from this file, in PyMOL (The PyMOL Molecular Graphics System, Version 2.3.2 Schrödinger) and used as the search model for molecular replacement using *phenix.phaser*[42], from the Phenix suite [41]. The NgSAT + l-Ser structure was solved in a similar manner but using an NgSAT (6WYE) monomer as the search model.

The models were initially built using the program *phenix.autobuild* [43] from the Phenix suite [41]. The resulting structure, was manually built and refined using COOT [44]. For manual building the 2*F*_o_–*F*_c_ and *F*_o_–*F*_c_ electron density maps, were set to 1σ and 3σ, respectively. Mobile sidechains without supported density were removed from models. Translation-liberation-screw (TLS) was carried out in the late stages of model refinement using TLS groups determined by *phenix.refine*. After each round of structure manipulation, *phenix.refine* was used to run rounds of real-space refinement [45].

### Ligand fitting and refinement

The structures were prepared for ligand fitting by building and refining the protein structure and adding in waters using *phenix.refine. Phenix.ligandfit* was used to search for ligands of interest [46,47]. Visual inspection and manual real-space refinement were carried out in COOT [44]. Metal ions were validated using the CheckMyMetal server [48].

### Structural analysis

Final structure statistics were generated using *phenix.table_one*, from the Phenix suite [41]. Structure images were generated using PyMOL (Schrödinger, LLC). The final co-ordinate and structure amplitude files for NgSAT and NgSAT + l-Ser were deposited to the PDB, under accession codes 6WYE and 7RA4, respectively.

### SAXS data collection

Measurements were performed at the Australian Synchrotron SAXS/WAXS beamline equipped with a Dectris-Pilatus detector. The wavelength of the X-rays was 1.0322 Å. The sample detector distance was 1426 mm. Data were collected from samples in a 1.5 mm thin-walled glass capillary at 25°C at two second intervals.

Size exclusion chromatography (SEC) in-line with the SAXS beamline (SEC-SAXS) [49] with a co-flow set-up [50] was used to collect scattering data. Data were collected from NgSAT and NgSAT + 50 µM l-cysteine following elution from a size exclusion column (Superdex 200 5/150), pre-equilibrated with SEC buffer (50 mM Tris pH 8.0, 100 mM NaCl) or SEC buffer with l-cysteine (50 mM Tris pH 8.0, 100 mM NaCl, 50 µM l-cysteine).

Raw scattering data were processed with Scatterbrain at the Australian Synchrotron. Scattered intensity (*I*) was plotted versus s using Primusqt from the ATSAS suite [51]. All samples were devoid of an increase in intensity at low s (indicative of aggregation). Guinier plots were linear for sRg < 1.15. Theoretical scattering curves were generated from the NgSAT crystal structure PDB file (6WYE) using Crysol [52].

## Results and discussion

### Purification and stoichiometry of NgSAT

The *N. gonorrhoeae* serine acetyltransferase (SAT) gene NGFG_RS07905 was cloned into pET28b for expression with an N-terminal His-tag. This was done to avoid adding residues onto the C-terminus of the protein which is proposed to interact with OASS/CysK in order to form the cysteine synthase complex in later studies. NgSAT was purified by IMAC followed by a final purification step via SEC. NgSAT eluted as a single peak from a size exclusion column with an elution volume of 12.56 ml corresponding to an approximate molecular mass of 193.8 kDa (Supplementary Figure S3). The predicted molecular mass of the NgSAT monomer is 31.6 kDa, confirming a hexamer of NgSAT monomers (6 × 31.6 kDa = 189.6 kDa). SDS–PAGE analysis of NgSAT shows >95% purity (Supplementary Figure S3).

### Kinetic properties of NgSAT

**Figure 1.**
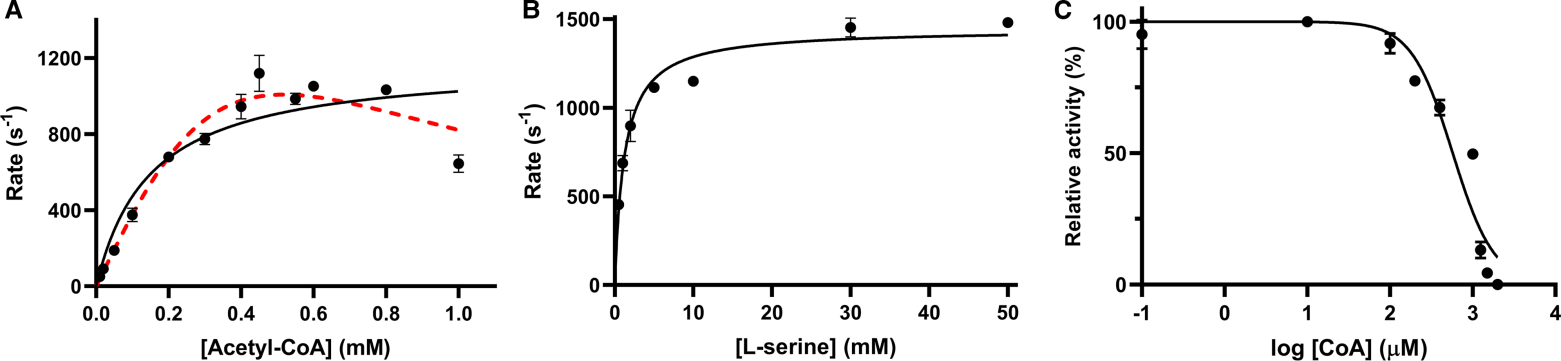
Kinetic analysis of NgSAT substrates l-serine and acetyl-CoA. (**A**) Substrate inhibition is observed for acetyl-CoA. Michaelis–Menten (black) and substrate inhibition equations (dashed red lines), are modeled. (**B**) Michaelis–Menten fit for l-serine. l-serine and acetyl-CoA assays were collected at saturating concentrations of 0.45 mM acetyl-CoA and 10 mM l-serine, respectively. Enzyme concentration was factored into the rates in (**A**) and (**B**) by dividing by the enzyme concentration to give the rate (s^−1^). (**C**) Dose response curve for coenzyme A (CoA). Line represents the fit of IC_50_ equation to data. Substrate concentrations were fixed at 0.15 mM and 1.5 mM, for acetyl-CoA and l-serine, respectively. Plotted data points represent mean alongside SEM of two replicates.

The activity of NgSAT was determined by measuring the decrease in absorbance at 232 nm due to depletion of substrate acetyl-CoA. The kinetic parameters for substrate acetyl-CoA (Table 1) were calculated from fitting a Michaelis–Menten curve (*R*^2^ = 0.8449) of rate (s^−1^; rate divided by enzyme concentration) versus acetyl-CoA (black line, Figure 1A). The overall fit for the Michaelis–Menten equation is reasonable with the exception of NgSAT rates collected at 1 mM acetyl-CoA which display inhibition that is likely to be substrate inhibition. We could not confidently fit the substrate inhibition equation (red line, Figure 1A) due to only one data point (1 mM acetyl-CoA) displaying inhibition. Due to limitations in experimental set-up (a high starting absorbance at high acetyl-CoA concentrations) we were unable to collect NgSAT rates for higher acetyl-CoA concentrations. However, data collected is highly reproducible with different enzyme preparations and enzyme concentrations (Supplementary Figure S4). From these data, the *K*_M (acetyl-CoA)_ was calculated to be 0.149 mM, and a *k*_cat_ of 1176 s^−1^ for the hexamer (Table 1).

**Table 1.** Kinetic parameters of NgSAT

Parameter	l-serine^a^	Acetyl-CoA^a^
*K*_M_ (mM)	1.21 ± 0.16	0.149 ± 0.05
*k*_cat_ (s^−1^)^b^	1444 ± 41	1176 ± 111
*k*_cat_/*K*_M_ (M^−1^.s^−1^)	1.19 × 10^6^ ± 0.16	7.89 × 10^6^ ± 2.54

a Error is SEM of two replicates;

b *k*_cat_ calculated by dividing the rate by enzyme concentration using the concentration of the NgSAT hexamer (189.6 kDa).

A concentration of 0.45 mM acetyl-CoA was the saturating concentration used for collection of the l-serine Michaelis–Menten plot as this from this point the rate plateaus (Figure 1A) and higher acetyl-CoA concentrations (∼1 mM) display likely substrate inhibition. The kinetic parameters for substrate l-serine were calculated from the Michaelis–Menten curve (*R*^2^ = 0.9476) of rate (s^−1^; rate divided by enzyme concentration) versus l-serine concentration (Figure 1B). The *K*_M (Ser)_ of the enzyme was calculated to be 1.21 mM, the *k*_cat_ 1444 s^−1^ for the hexamer (Table 1).

To investigate if NgSAT is inhibited by the high concentrations of the reaction product CoA as observed for other SAT enzymes [29,30] we collected an IC_50_ inhibition curve for CoA. There is inhibition of NgSAT activity with increasing concentrations of CoA (Figure 1C) indicating product inhibition at high CoA concentrations with an IC_50_ value of 573 ± 11 µM, and a hillslope of −1.7 ± 0.3 indicating positive cooperativity. While CoA exhibits product inhibition, it does not account for the decrease in rate seen at 1 mM acetyl-CoA (Figure 1A) as reaction rates were calculated from steady-state conditions.

The difference in *K*_M_ values for each substrate (1.21 mM l-serine versus 0.149 mM acetyl-CoA), demonstrates a greater affinity of NgSAT for acetyl-CoA compared with l-serine, which is consistent with other SAT homologues [6,29,30]. The specificity constants, *k*_cat_/*K*_M_, for NgSAT are 1.19 × 10^6^ and 7.89 × 10^6 ^M^−1^.s^−1^ for l-serine and acetyl-CoA, respectively, which are below the diffusion theory maximum rate of ∼10^8 ^M^−1^.s^−1^, indicating that the enzyme rate is not diffusion limited [54].

There is a 6.5-fold increase in *k*_cat_/*K*_M_ for acetyl-CoA compared with l-serine attributable to NgSAT having a greater affinity for acetyl-CoA, with an approximately eight-fold lower *K*_M_ for acetyl-CoA (0.149 mM) compared with l-serine (1.21 mM). SAT enzymes from *E. coli* and *H. influenzae* display Michaelis–Menten kinetics for acetyl-CoA, but exhibit substrate inhibition for l-serine [5,12,30]. NgSAT appears to be the opposite, where acetyl-CoA possibly exhibits substrate inhibition and l-serine does not. Based on the reaction mechanism of SAT homologues [12,30], we predict that NgSAT has a sequential mechanism, in keeping with the proposed formation of the ternary complex during acyl transfer to l-serine.

The acetyl-CoA binding site of NgSAT, may prove to be a good target for inhibitors, and this is being explored in other hexapeptide transferases. Inhibitors for a related hexapeptide transferase termed GlmU targeted the acetyl-CoA binding site, instead of the active site (NgSAT equivalent l-serine binding site) [54].

### Structure of NgSAT

**Figure 2.**
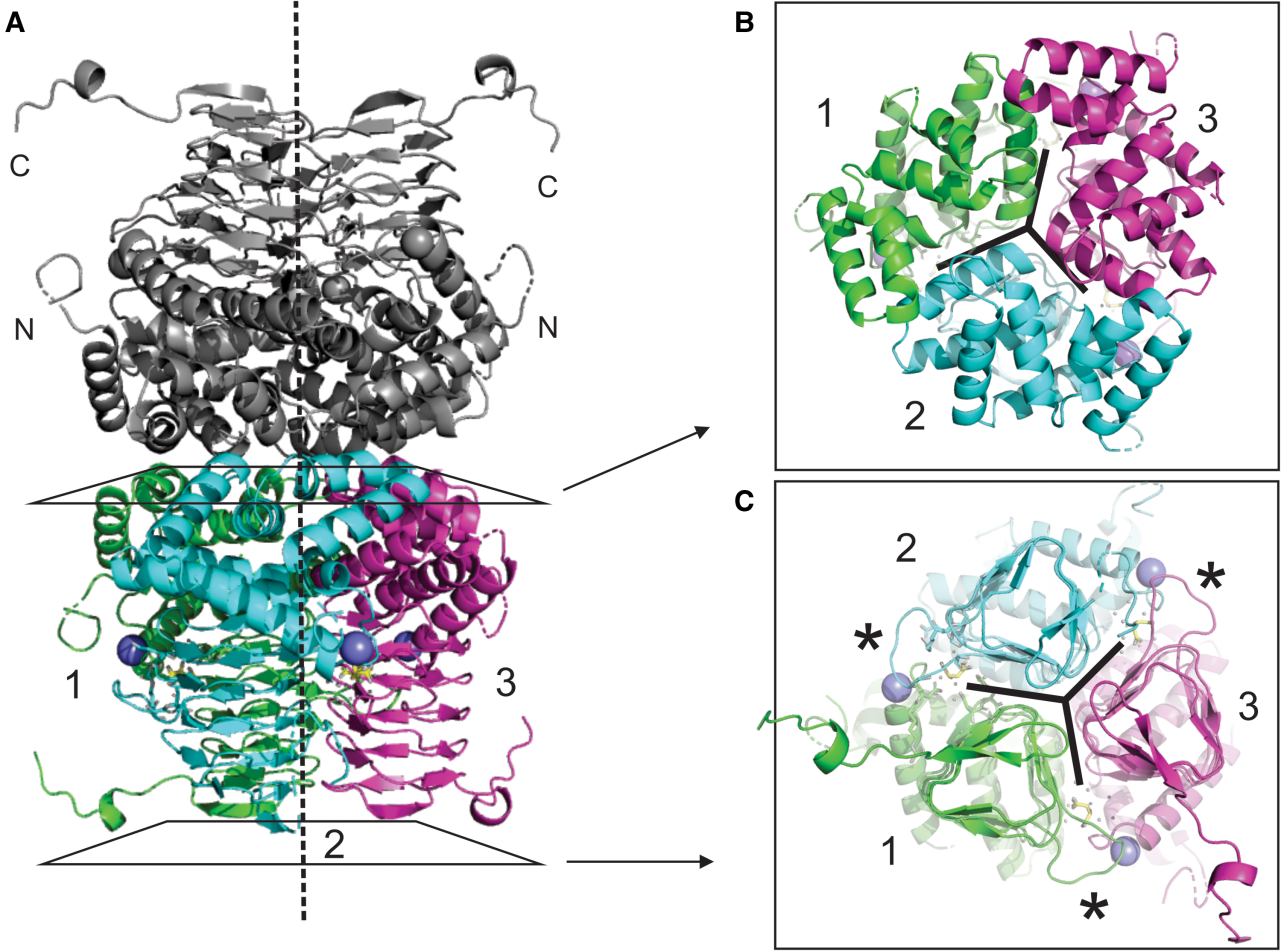
Structure of the NgSAT hexamer. A ribbon diagram of the overall structure of NgSAT shows that a dimer of trimers forms the hexameric unit through interactions between the N-terminal α-helical domains (**A**). Trimers form through interactions of the N-terminal α-helices (**B**) and left-handed β-helical C-terminal domain (**C**). The vertical dashed line represents the independent three-fold symmetry which is evident in (**B**) and (**C**). Sodium ions are shown as a purple spheres and l-malate is shown as yellow sticks. Asterisks shows active site extended loop. This figure was prepared with PyMOL.

The crystal structure of NgSAT was solved by molecular replacement to 2.01 Å with l-malate (from the crystallization condition) bound in the active site. The final model was refined to an *R* and free *R* of 0.183 and 0.222, respectively (Table 2). The asymmetric unit contained six monomers arranged as one complete trimer and another trimer split into 1/3 and 2/3. A complete hexameric molecule is apparent after repetition by a crystallographic two-fold axis (Figure 2A). The most complete subunits contained residues 3-264 of the 272-residue polypeptide. Electron density corresponding to the uncleaved 21-residue N-terminal histidine tag was not apparent for any subunit. The C-terminal tail adopts two conformations, with the tails of two monomers in the trimer extending away from the trimer and the remaining monomer exhibiting a folded tail conformation. The two monomers in the hexamer (one from each trimer) with folded C-terminal tails had poor electron density for residues 250–254 towards the C-terminal end. Data show the presence of l-malate (present in the crystallization condition) forming meaningful active site interactions in the structure.

**Table 2.** Data collection and refinement statistics

	NgSAT	NgSAT + l-Ser
*Data collection* ^a^		
Wavelength (Å)	0.9537	0.9537
Resolution range (Å)	43.11–2.01 (2.08–2.01)	43.88–2.80 (2.8–2.95)
Space group	*P*2_1_	C2
Unit cell parameters *a*, *b*, *c* (Å)	77.90, 93.87, 102.02,	102.86, 94.59, 79.25
*α*, *β*, *γ* (°)	90.00, 91.29, 90.00	90.00, 92.18, 90.00
No. of molecules in asymmetric unit	6	3
Total reflections	689 150 (70 393)	130 367 (19 576)
Unique reflections	97 700 (9715)	18 772 (2723)
Multiplicity	7.1 (7.2)	6.9 (7.2)
Completeness (%)	99.6 (100.0)	99.9 (100.0)
Mean *I*/*σ*(*I*)	18.5 (3.4)	9.1(2.2)
*R*_merge_^b^	0.05574 (0.4889)	0.149 (0.915)
*Refinement*		
Reflections used in refinement	97 327 (9715)	130 367 (19 576)
Reflections used for *R*_free_	4749 (424)	18 772 (2723)
*R*_work_	0.1830 (0.2178)	0.2346 (0.2892)
*R*_free_	0.2219 (0.2745)	0.2673 (0.3240)
No. protein atoms	11 158	5001
No. solvent atoms	538	39
No. ligand atoms		
l-malate	54	—
Sodium	6	—
l-serine	—	14
Protein residues	1534	726
r.m.s.d bonds (Å)	0.007	0.002
r.m.s.d angles (°)	0.84	0.59
Ramachandran favored (%)	97.7	95.61
Ramachandran allowed (%)	2.3	4.39
Ramachandran outliers (%)	0	0
Average *B* (Å^2^)	38.9	54.19
*Macromolecules*	38.8	54.20
*Ligands*	37.0	58.56
*Solvent*	40.3	50.78
Clashscore	2.3	2.25
No. of TLS groups	1	4
PDB entry	6WYE	7RA4

a Statistics for highest resolution shell are shown in parentheses;

b $R_{\mathrm{merg}}=\sum_{\;j=1}\left|I_{hkl,j}-\langle I_{hkl}\rangle \right|/\underset{hkl}{\sum }\underset{j}{\sum }I_{hklj}$.

The crystal structure of NgSAT with serine bound (NgSAT + l-Ser) was solved using a closed tail monomer of NgSAT (6WYE) and was refined to 2.8 Å. The model has an *R* and free *R* of 0.234 and 0.267, respectively. A trimer is present in the asymmetric unit but a hexamer can be generated using the crystallographic two-fold axis. There was density to support placement of substrate l-serine in two of the three active sites, with the third having weak density, where serine interacts with conserved active site residues.

There are no major conformational differences between the l-malate and l-serine bound structures (RMSD 0.236 Å), where the main differences are due to the presence of open tail conformations in NgSAT. Density is present for extended and folded C-terminal tails in the NgSAT + l-Ser structure (one closed and two open tails), but as a result of poorer resolution for this structure it was not possible to build the tails into the density. Given that the NgSAT structure has better resolution, it was used for downstream structural analysis.

**Figure 3.**
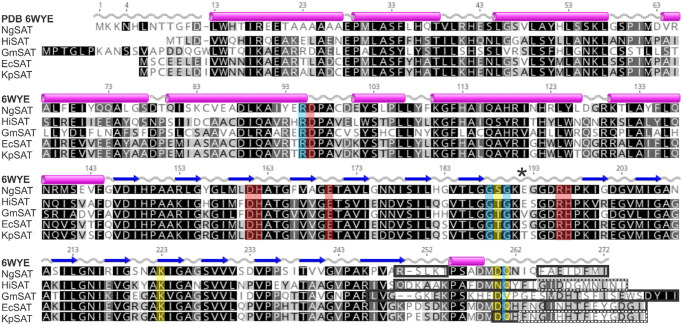
Sequence alignment of SAT enzymes. An amino acid sequence alignment of the most structurally similar SATs to *N. gonorrhoeae* shows strict conservation of residues in the C-terminal LβH domain and in the active site. Secondary structure and numbering presented is from *N. gonorrhoeae* (6WYE) showing α-helices as pink cylinders and β-strands as blue arrows. Letter shading is based on % similarity. Red shading shows active site residues and blue shading shows additional important active site solvent interacting residues. Yellow shading shows residues identified in C-terminal tail folding and black (and dashed) boxes illustrate missing residues from these structures. *N. gonorrhoeae* (NgSAT; AAW90067.1), *H. influenzae* (HiSAT; WP_005694542.1), *G. max* (GmSAT; XP_003528805.2), *E. coli* (EcSAT; WP_114569552.1) and *K. pnemoniae* (KpSAT; Q0ZB96). Asterisk shows the active site extended loop region. This figure was prepared with Geneious Prime 2019.2.1.

The SAT monomer can be subdivided into an N-terminal domain (residues 1–144) consisting of eight α-helices and a C-terminal left-handed β-helical domain (LβH; residues 145–272). Three monomers interact with each other through contacts between helices in the N-terminal domain and interactions of the LβH domain to form the trimer, which in turn form the hexamer as a dimer of trimers (Figure 2). The overall fold of the NgSAT monomer and its hexameric state, is similar to the bacterial and plant homologues and strict residue conservation between species indicates the significance of this type of fold in SATs ([8,13,14], Figure 3).

**Figure 4.**
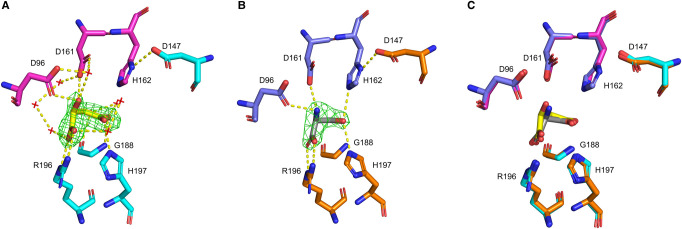
Simulated-annealing OMIT maps of ligand bound active sites for NgSAT and NgSAT + l-Ser. The placement of l-malate (yellow sticks) in the NgSAT active site (adjacent monomers colored magenta and blue) (**A**) and l-serine (gray sticks) in the NgSAT + l-Ser structure (adjacent monomers colored purple and orange) (**B**) are supported by *F*_o_–*F*_c_ maps displayed as green mesh (contoured at 3.0σ). (**C**) Overlay of NgSAT and NgSAT + l-Ser monomers (open conformation) demonstrates conserved orientation of active site residues Asp96, Asp147, Asp161, His162, Gly188, Arg196 and His197 (displayed as sticks). Hydrogen bonds between active site residues and ligands are represented by yellow dashed lines. Waters are shown as red crosses in (**A**) no waters are shown in (**B**) due to the difficult placement of waters due to resolution. This figure was prepared using PyMOL.

**Figure 5.**
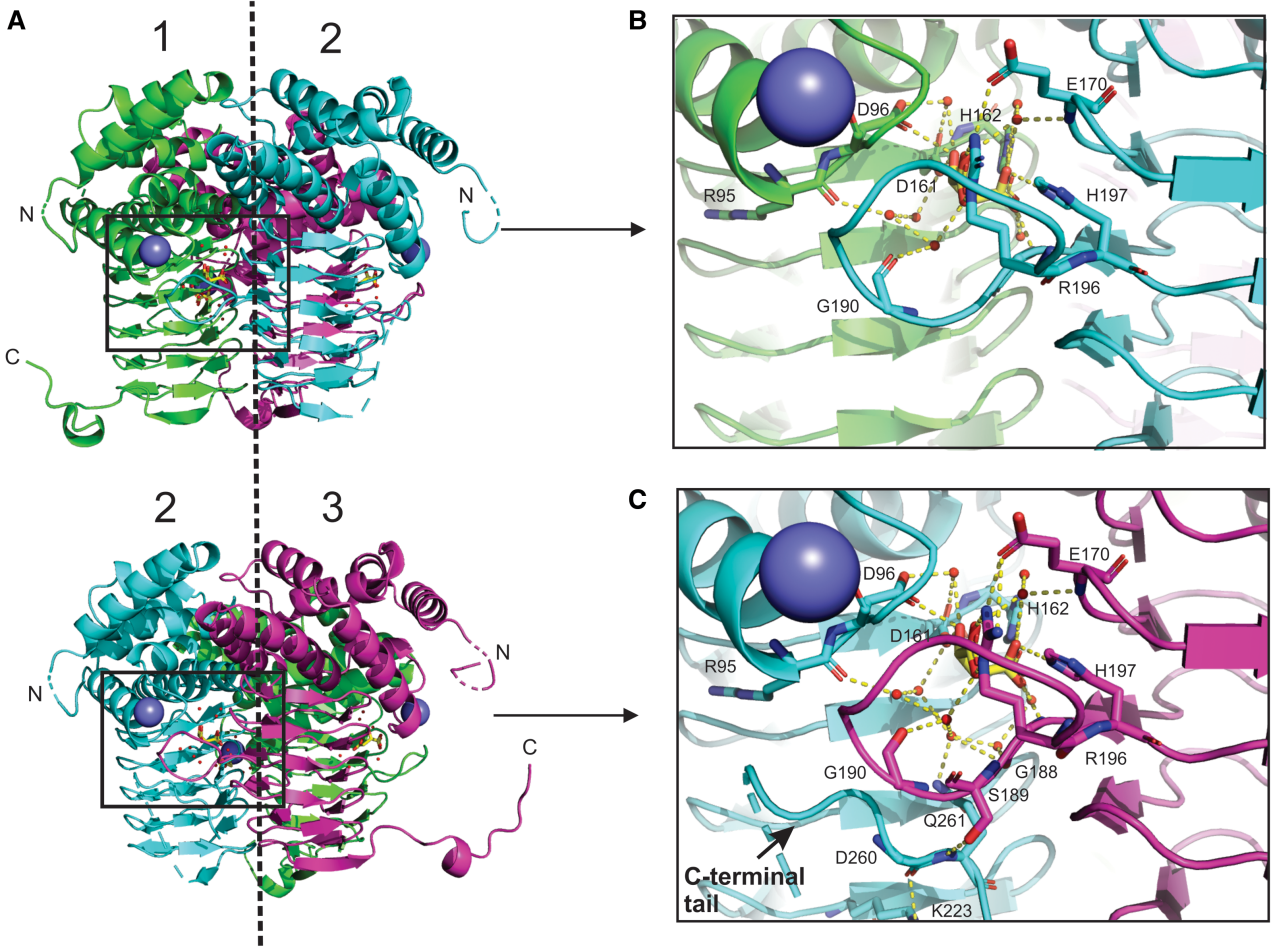
A ribbon diagram of the NgSAT trimer illustrates the position and formation of the active site between two adjacent monomers. The structure reveals different C-terminal tail conformations in different active sites of a single trimer (**A**). Between monomers 1 and 2 the C-terminal tail of monomer 1 (green) is extended away from the active site (**B**). Between monomers 2 and 3 the C-terminal tail of monomer 2 (blue) is tucked up into the active site (**C**). Sodium ions are shown as a purple spheres and l-malate is shown as yellow sticks. Dashed line represents the three-fold symmetry. This figure was prepared with PyMOL.

The acetyltransferases are characterized by a unique hexapeptide repeat ([LIV]-[GAED]-X_2_-[STAV]-X) which results in the formation of the LβH domain. There is a single deviation from the LβH domain structure between β-strands 7 and 8, which creates an extended loop (residues 191–199 in this structure) which is significant as this forms one half of the active site (Figures 2C and 4). The active site is situated between the C-terminal domains of two adjacent monomers. In each trimer there are three active sites. In a trimer positioned with the N-terminus top and C-terminus bottom, the active site is located at the intersection of the N-terminal domain and C-terminal domain of the left monomer and the inner face of the extended loop from the right monomer (Figure 5). The six active site residues in *N. gonorrhoeae* comprise of Asp96, Asp161 and His162 from the left monomer and Asp147, Glu170, Arg196 and His197 from the right monomer (Figures 3 and 4), with His162 and Asp147 forming the catalytic dyad.

Comparing the two NgSAT structures, the active site residues, Asp96, Asp161, Arg196 and His197 (Figure 4), interact with l-malate in a similar manner as the substrate l-serine. The terminus carboxylic acid group on l-malate and l-serine are co-ordinated by the Arg196 side chain. The negatively charged carboxylate side chains from residues Asp96 and Asp161 both co-ordinate the l-serine backbone amide and the hydroxyl group in l-malate (Figure 4A). The most notable difference in active site coordination is where the l-serine γ hydroxyl forms meaningful hydrogen bonds to flanking His162 and His197 (Figure 4B), whereas in the NgSAT malate bound structure the equivalent carboxylic acid group maintains a hydrogen bond with His197 but is orientated away from the catalytic His162, and is co-ordinated by the backbone amide of Gly188 (Figure 4). This difference is likely due to l-serine being a natural substrate of the enzyme, whereas l-malate is not. The interaction of l-serine with active site residues in the NgSAT + l-Ser structure is similar to the other l-serine bound SAT structure in the PDB (4N69) from Soybean (*Glycine max*) [24], with the exception that His162 is interacting with l-serine in the NgSAT + l-Ser structure.

The C-terminal tail adopts two conformations in our malate bound structure. For two monomers, the tails extends outward away from the trimer, reaching towards adjacent trimers in the crystal unit and occupying the interface between two monomers (exhibiting domain swapping). For the remaining single monomer in each trimer, the tail loops upward against the side of the monomer and tucks under the active site (Figure 5). There are notable differences in the number of water molecules participating in the active site chemistry with l-malate when the tail is positioned near the active site (Figure 5B,C). In the extended tail conformation with an ‘open’ active site there are seven water molecules interacting with l-malate, whereas in the tucked tail conformation with a ‘closed’ active site there are an additional two water molecules coordinating with l-malate (Figure 5B,C). The ‘closed’ active site recruits additional residues Gly188 and Gln261 to join Arg95 and Gly190 in solvent interactions and residues Ser189, Lys223, Asp260 and Gln261 become involved in tail interactions (Figure 5C). Glutamine 261 is noted to be of particular importance in this ‘closed’ active site conformation as this has key interactions securing the tail position and involvement in the active site chemistry, drawing in additional water molecules. Notably the l-malate bound structure presented here has only eight missing C-terminal residues compared with related structures (Figure 3) that have more missing residues. When the active site is in the ‘closed’ conformation there is no access to the interior serine binding pocket.

The tucked tail conformation of the NgSAT monomer is identical with homologous SAT structures that have the product cysteine bound (PDB 1SSQ, 1T3D, 3GVD, 3P47, 4H7O and 6JVU) and analysis of these structures also reveals that there would be no access to the pocket of the active site (regardless of the number of missing C-terminal residues). Due to an artifact of crystallographic packing, we have surprisingly been able to examine two active site states regardless of having a true substrate molecule bound. l-malate was present in the crystallization condition and its occupation of the active site is expected to be based on charge in a similar manner to the inhibitor l-cysteine. Comparing the ‘closed’ active site of NgSAT with the homologous *E. coli* SAT (1T3D) (containing cysteine in the active site) we observe a similar arrangement of active site residues and the C-terminal tail position. Cysteine acts as a negative feedback regulator of SAT activity and it could be deduced that it does this by ‘closing’ the active site. Comparing the ‘open’ active site of NgSAT with the *H. influenzae* homologue (1SST) (containing CoA in the active site) we observe that the C-terminal tail is also orientated away from the active site and in addition the extended loop in 1SST is disrupted whereas in our structure is not. The substrate CoA is a large molecule and by occupying the active site the C-terminal tail cannot fold into this space. The structure presented here is unique in that we see both ‘open’ and ‘closed’ conformations in a monomer which gives insight into substrate binding.

### Cysteine inhibition

**Figure 6.**
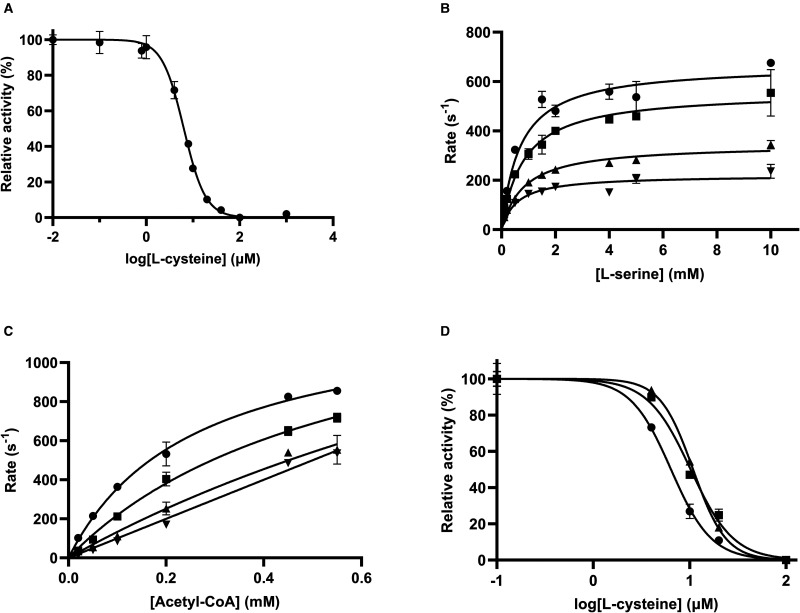
Characterization of cysteine inhibition. (**A**) Dose response curve for l-cysteine. Line represents the fit of IC_50_ equation to data. Substrate concentrations were fixed at 0.15 mM and 1.5 mM, for acetyl-CoA and l-serine, respectively. (**B**) Michaelis–Menten plots were collected for 0 (circles), 4 (squares), 6 (triangles) and 8 (inverted triangles) µM of l-cysteine, with varying concentrations of serine. (**C**) Michaelis–Menten plots for 0 (circles), 4 (squares), 6 (triangles) and 8 (inverted triangles) µM of l-cysteine, with varying concentrations of acetyl-CoA (x-axis). (D) Dose–response curves for l-cysteine in the presence of 0.15 (circles), 0.25 (squares) and 0.45 (triangles) mM of acetyl-CoA. Data points are mean and error bars are SEM derived from two replicates.

For SAT enzymes, feedback inhibition by l-cysteine is well-documented across both bacterial and plant species [5,30,55]. Here, we characterized l-cysteine inhibition of NgSAT, relative to the substrates l-serine and acetyl-CoA. NgSAT activity was inhibited by increasing concentrations of l-cysteine with an IC_50_ of 6.4 ± 0.1 µM and a hill slope of −1.86 ± 0.15 (Figure 6A). Collection of Michaelis–Menten plots with varying l-serine concentrations (Figure 6B) in the presence of fixed concentrations of cysteine, increased the *K*_M(serine)_ from 1.16 mM with no l-cysteine bound to 4.83 mM in the presence of 8 µM l-cysteine, while the *k*_cat_ did not change. This demonstrates that l-cysteine is a competitive inhibitor relative to l-serine. Competitive inhibition is supported by structural evidence from *E. coli* and *E. histolytica* where cysteine has been shown to bind directly to the l-serine active site, preventing l-serine from binding [6,27]. We could not confidently fit competitive, non-competitive or uncompetitive enzyme inhibition models to the data. Incorporation of the hill coefficient (Supplementary equation 1) [35,36] into the competitive inhibition model accounts for cooperativity of binding and is well accounted for in the data (Supplementary Figure S5). The *h* value (a measure of cooperativity) decreases steadily from 1 (non-cooperative binding) to 0.299 (negatively cooperative binding) with increasing l-cysteine concentrations (Supplementary Table S1), suggesting negative cooperativity between active sites when l-cysteine is present. This is consistent with the hill slope (−1.86) of the l-cysteine dose response curve (Figure 6A), where binding of l-cysteine increases the affinity of other active sites in the multimer for l-cysteine (positive cooperativity) which reduces the affinity for l-serine (negative cooperativity in Figure 6B and Supplementary Figure S5). Although cooperativity has not been reported for SAT homologues, together with the positive cooperativity seen in the CoA dose response curve (Figure 1C), it clear that there is interconnectedness between active sites in NgSAT, which is unsurprising given the multimeric nature of the enzyme.

l-cysteine has also been shown to be a competitive inhibitor relative to acetyl-CoA in SAT enzymes from other bacteria [6,27]. To investigate if l-cysteine is a competitive inhibitor relative to acetyl-CoA in NgSAT we collected Michaelis–Menten plots with varying acetyl-CoA concentrations (Figure 6C) in the presence of 4, 6 and 8 µM l-cysteine respectively. We were unable to confidently determine the mode of inhibition, with both competitive and non-competitive inhibition models fitting the data reasonably. Incorporation of the hill coefficient into either equation did not improve the fit of either model. Collection of IC_50_ curves in the presence of increasing concentrations of acetyl-CoA (Figure 6D), demonstrate an increase in IC_50_ from 6.3 µM (hill coefficient 2.1 ± 0.2) to 10.8 µM (hill coefficient 2.5 ± 0.1), consistent with a competitive inhibition mechanism [57] and positive cooperative for l-cysteine binding. Competitive inhibition is supported by the observation that in cysteine-bound SAT structures from other bacteria, the flexible C-terminal tail of SAT is folded against the active site [6,23,27], as also seen in the ‘closed’ active sites in our NgSAT structure. This essentially closes the active site, preventing acetyl-CoA from binding, thereby acting as a competitive inhibitor.

### Small angle X-ray scattering shows a conformational change in the presence of l-cysteine

**Figure 7.**
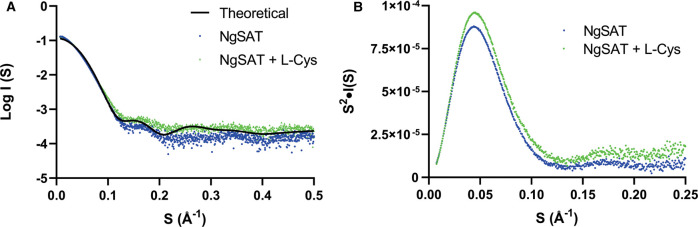
SAXS profiles of NgSAT bound and unbound to l-cysteine. (**A**) SAXS profiles of NgSAT were measured in the absence of (blue circles) and presence (green circles) of 50 µM l-cysteine (l-Cys). Theoretical scattering (black line) calculated from cysteine-free NgSAT crystal structure. (**B**) Kratky plot of NgSAT in the absence (blue circles) and presence (green circles) of 50 µM l-cysteine.

SEC in line with small angle X-ray scattering (SEC-SAXS) analysis [50] was performed to determine the effect of l-cysteine binding to NgSAT in solution. Crystallization attempts of NgSAT with l-cysteine were unsuccessful due to degradation of crystals upon addition of l-cysteine. The scattering data were consistent with a globular protein (Figure 7), and the calculated molecular mass based on the Porod volumes, combined with the pair wise distribution (P(*r*)) analysis support a hexamer of NgSAT monomers. The calculated molecular mass for the l-cysteine bound and unbound structures was the same (193.87 kDa). The molecular mass of the monomer including the His-tag is 31.6 kDa, therefore, 193.87 kDa divided by the molecular mass of the monomer (31.6 kDa) is 6.1 monomers per structure, consistent with the hexameric crystal structure and SEC presented above. The SAXS profiles were fit with the calculated theoretical scattering from our NgSAT crystal structure. The theoretical scattering profile shows a poor fit with the scattering data collected for unbound (*χ*^2^ = 5.31) and l-cysteine bound NgSAT scattering data (*χ*^2^ = 10.84) (Figure 7A), which is not unexpected due to combination of closed (tucked C-terminal tail) and open (flexible C-terminal tail) conformations seen in the NgSAT crystal structure which the theoretical scattering is calculated from.

Comparison of the SAXS profiles in the presence and absence of l-cysteine shows clear differences (Figure 7A), consistent with a conformational change upon binding of l-cysteine. The difference is associated with an increased compactness of the enzyme when bound to l-cysteine as shown by the smaller radius of gyration derived from the Guinier Plot (*Rg* = 40.26 ± 0.35 Å with l-cysteine bound compared with *Rg* = 43.01 ± 0.27 Å without) and P(*r*) analysis (*Rg* = 40.8 ± 0.14 Å for l-cysteine bound compared with *Rg* = 43.17 ± 0.14 Å for unbound). The scattering data, including the pairwise distribution analysis and *Rg* values from the Guinier analysis are consistent with SAXS data from SAT from *E. coli* [58].

The bell-shaped curve observed in the Kratky plot (Figure 7B) shows that the protein exhibits a typical folded shape. This confirms the protein remains folded in the presence of l-cysteine although the cysteine-free curve is slightly flattened suggesting slightly increased flexibility of the protein. Decreased flexibility and an increased compactness of the cysteine bound enzyme is consistent with previously reported SAT structures crystallized with the cysteine inhibitor [6,23,27]. The flexible C-terminal tail of SAT in cysteine bound structures adopts a more rigid, less flexible structure by interacting with the third β-coil to bury the acetyl-CoA binding site [27] which is also consistent with the ‘closed’ state for NgSAT that we see in our crystal structure leading to small conformational changes and a slightly more compact enzyme.

## Conclusions

Cysteine synthesis in bacteria is a two-step reaction, the first step catalyzed by SAT that produces *O-*acetylserine from l-serine and acetyl-CoA, and the second step catalyzed by *O*-acetylserine sulfhydrylase (OASS) that uses the substrates sulfide or thiosulfate to synthesize l-cysteine from *O-*acetylserine. The SAT and OASS-A enzymes form the cysteine synthase complex with the C-terminal tail of SAT binding into and inhibiting the active site of OASS-A. SAT is feedback inhibited by the end product of the pathway l-cysteine. Bacterial members of the SAT family are generally hexamers (with the exception of *E. histolytica* [27]) and are characterized by a unique left-handed parallel β-helix (LβH).

Here we have presented structural and kinetic data for SAT from *N. gonorrhoeae* (NgSAT). SEC and small angle X-ray scattering (SAXS) analysis confirm that NgSAT is a hexamer in solution as well as in the crystal structure, with no other oligomeric states detected. We have characterized the kinetics of NgSAT, and hypothesize a sequential mechanism, although the order of substrate binding is unknown. We suggest substrate inhibition at concentrations of acetyl-CoA ≥ 1 mM but due to limitations in experimental set-up were not able to confirm substrate inhibition. This is in contrast with other SAT enzymes from other bacteria including *E. coli* and *H. influenzae* that display substrate inhibition by l-serine [6,23]. NgSAT also exhibits product inhibition by coenzyme A (CoA) consistent with other SAT enzymes [29,30]. Cooperativity is seen in the presence of the inhibitor l-cysteine and the product CoA indicating an interconnectedness between active sites in the NgSAT hexamer. NgSAT is feedback inhibited by the end product of the pathway l-cysteine, and using SAXS we show small conformational changes in the cysteine bound enzyme. These changes suggest a less flexible, slightly more compact enzyme when bound to cysteine. This is consistent with the ‘closed’ active sites we see in the NgSAT crystal structure and crystal structures of other SAT enzymes bound to l-cysteine [6,23,27], where the C-terminal tail is tucked up against the side of the active site in the closed (cysteine-bound) structures leading to a more rigid enzyme.

The structure of NgSAT reveals that this enzyme is clearly part of the SAT family due to its hexameric nature and LβH. As with other related structures, NgSAT is a dimer of trimers, with each monomer comprising an N-terminal α-helical domain and a C-terminal LβH domain. The majority of SAT three-dimensional structures are incomplete and have up to 37 amino acids missing from the C-terminal end of the enzyme. We note that the NgSAT structure has the least number of missing residues from the distal C-terminal end compared with related structures, which has allowed us to investigate the topology of the active site with some confidence.

A crystallographic artifact has led to two different C-terminal tail conformations in the NgSAT structure. Where other structures have missing residues, leading to an exposed active site cavity, NgSAT exhibits either a free C-terminal tail swept outwards from the enzyme or a constrained C-terminal tail tucked up into the active site. The outward C-terminal tail results in an ‘open’ active site, in contrast with the tucked tail which results in a ‘closed’ active site. The ligand l-malate is present in the active site of NgSAT due to its presence in the crystallography reagent. It occupies the same area and interacts with the active site residues that the natural substrate l-serine would, as seen in our NgSAT + Ser structure, with the exception of catalytic His162.

The tucked C-terminal tail ‘closed’ conformation reveals interesting interactions with both the solvent and other residues. A key glutamine residue (Gln261) in the tail, interacts with two additional water molecules in the active site (assisted by a nearby Gly188) that were otherwise uninvolved in the ‘open’ conformation. The interaction of Gln261 in the C-terminal tail brings a serine residue near the active site into close-range to allow it to form polar contacts with an aspartate on the C-terminal tail, which in turn forms a polar contact with a lysine from a β-strand in the LβH. Analysis of the residues involved in these polar contacts to tuck the C-terminal tail into the active site reveals strict conservation between species. Overall, there is a chain reaction of polar contacts from the active site, downwards through the tail, securing it and closing the active site.

In bacteria the primary pathways of inorganic sulfur assimilation converge at cysteine biosynthesis and *N. gonorrhoeae* lacks the ability to utilize sulfate as an inorganic sulfur resource, but its requirement for sulfur can be fulfilled by thiosulfate [10]. Curiously *N. gonorrhoeae* lacks the variant of the OASS enzyme that utilizes thiosulfate (OASS-B/CysM), having just a single OASS enzyme with homology to OASS-A/CysK (the variant that utilizes sulfide not thiosulfate) [9]. It remains to be seen if this enzyme does indeed utilize only sulfide as a substrate or if it is a dual-function enzyme, also capable of using thiosulfate for the synthesis of cysteine. In addition it is unknown if OASS-A from *N. gonorrhoeae* interacts with NgSAT to form the cysteine synthase complex although it is likely, as the C-terminal tail of NgSAT contains the conserved terminal isoleucine critical for binding of the tail into the active site of OASS [13,14,58].

Our NgSAT structures presented here, alongside kinetic and inhibition data, gives unique insight into the structure, function and inhibition of SAT from *N. gonorrhoeae* and due to the unique nature of sulfur acquisition for cysteine biosynthesis in *N. gonorrhoeae* could represent a novel drug target for treating extensively antimicrobial resistant gonorrhoea.

## Associated content

UniProt Accession ID for NGFG_01496 (NgSAT): Q5F6X0 (Q5F6X0_NEIG1)

## Data Availability

The final co-ordinate and structure amplitude files for NgSAT and NgSAT + l-Ser are deposited in the Protein Data Bank (https://www.rcsb.org/) under PDB accession codes 6WYE and 7RA4, respectively.

## Abbreviations

Acetyl-CoA: acetyl coenzyme A
CoA: coenzyme A
IMAC: immobilized-metal affinity chromatography
IPTG: isopropyl β-d-thiogalactopyranoside
LB: Luria−Bertani
l-cys: l-cysteine
l-Ser: l-serine
LβH: left-handed β helix
NgSAT: *Neisseria gonorrhoeae* serine acetyltransferase
OAS: *O*-acetylserine
OASS: *O*-acetylserine sulfhydrylase
SAT: serine acetyltransferase
SAXS: small angle X-ray scattering
SEC: size exclusion chromatography

## Supplementary Material

Supplementary Figures 1-5, Equation 1 and Table 1
