# Supplementary material for: Serine acetyltransferase from Neisseria gonorrhoeae; structural and biochemical basis of inhibition
Source: Biochem J. 2022 Jan 6;479(1):57–74. doi: 10.1042/BCJ20210564 (PMC8786284; doi:10.1042/BCJ20210564)
Supplement: Supplementary Figures 1-5, Equation 1 and Table 1 [file BCJ-479-57-s1.pdf]

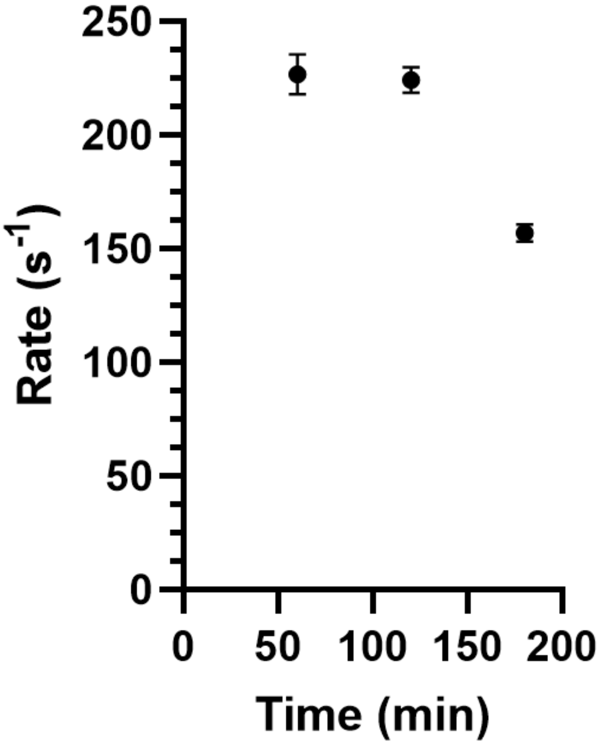

Supplementary Figure 1. *NgSAT activity versus time post-purification. NgSAT was stored at room temperature post-purification and activity measured every 60 minutes (x-axis). Assays were collected with 0.625 µg NgSAT, 1 mM serine and 0.45 mM acetyl-CoA. Data points are mean and error bars are SEM derived from two replicates.*

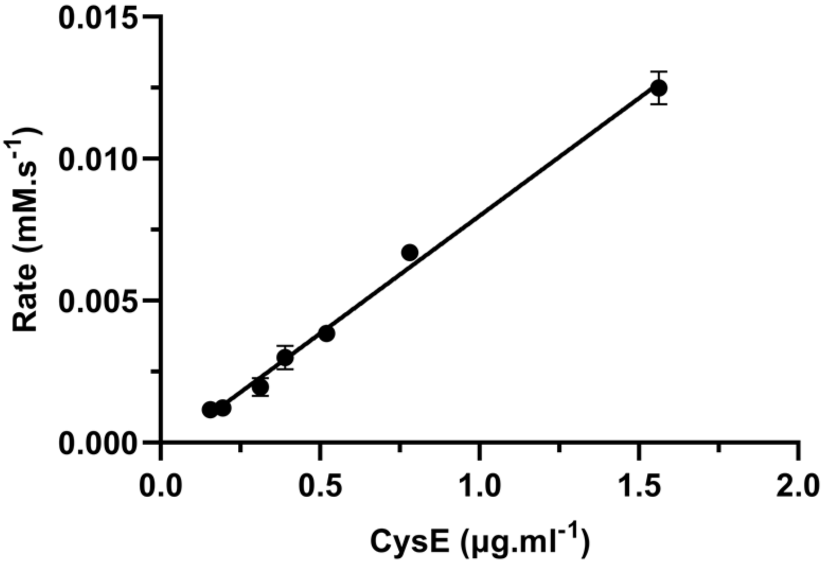

Supplementary Figure 2. *NgSAT activity measured at varying NgSAT concentrations. Activity was measured for 0.156, 0.312, 0.391, 0.521, 0.781 and 1.56 µg.ml^{-1} of NgSAT (x-axis). Initial rates were measured in assay buffer in the presence of 0.45 mM acetyl-CoA and 10 mM L-serine. Enzyme stocks were stored at room temperature (22°C) for the duration of the assays. Data points are means derived from duplicates and standard error is displayed as error bars.*

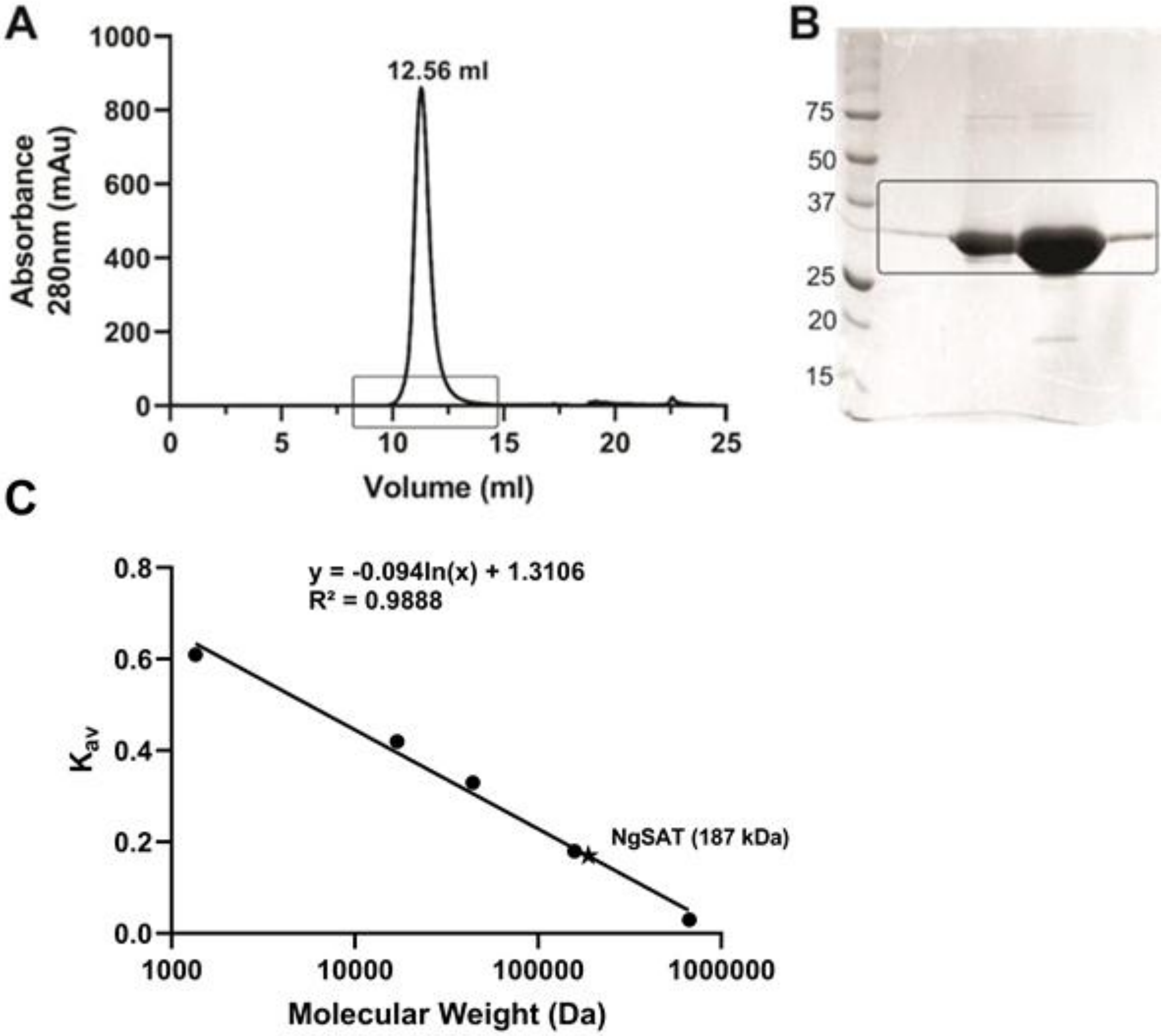

Supplementary Figure 3. *Gel filtration purification of NgSAT. (A) Gel filtration chromatogram of purified NgSAT using an Enrich 650 Gel Filtration Column (Bio-Rad) with a single elution peak at 12.56 ml. (B) Corresponding 12% SDS-PAGE gel of column fractions (outlined by a box on the chromatogram and the SDS-PAGE gel) showing a high yield and purity of NgSAT. Molecular weights of Precision Plus Protein Standards (Bio-rad) in kDa are labelled. (C) Calibration of the Enrich 650 Gel Filtration Column. Molecular weights of gel filtration calibration standards (Bio-Rad) plotted on the x-axis. Kav plotted on the y-axis. Kav of NgSAT (0.17) indicated by star. Kav calculated by subtracting column void volume from the protein elution volume and dividing by the total column volume. Equation from line of best fit used for determining molecular weight of unknown proteins.*

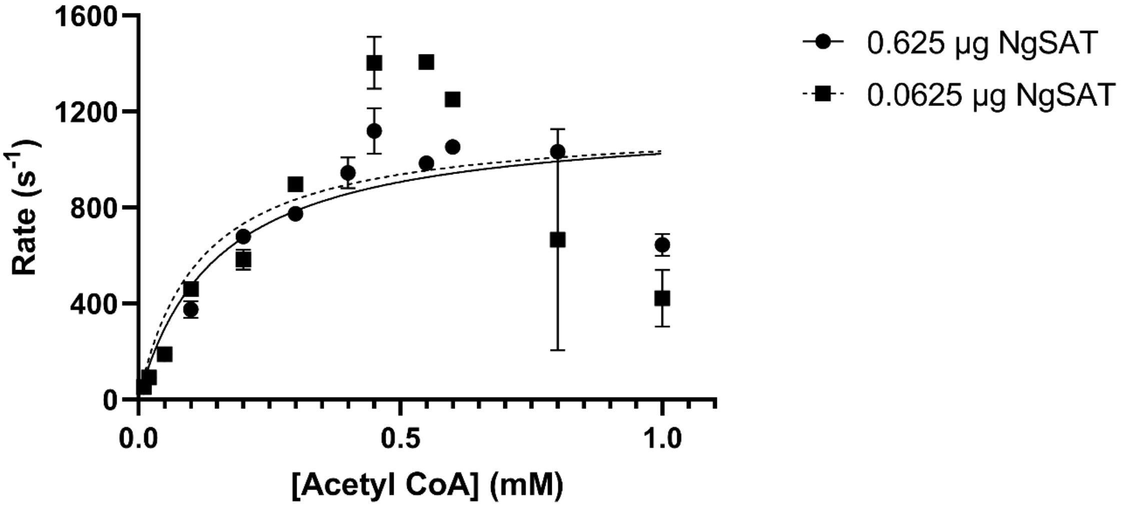

Supplementary Figure 4. *Kinetic analysis of NgSAT substrate acetyl-CoA with two different concentrations of NgSAT (0.625 and 0.0625 µg) at 10 mM L-serine. Although reproducible at two different concentrations, with two different enzyme preparations, data for the 10-fold lower NgSAT concentration (0.0625 µg) show increased error due to fluctuations in the data at higher starting absorbance values due to high acetyl-CoA concentrations. The Michaelis-Menten equation is fit (solid line represents 0.625 µg NgSAT (R^2 = 0.8449) and dashed line represents 0.0625 µg NgSAT (R^2 = 0.5240)) for both data sets and shows reproducibility. The overall fit for the equation is reasonable, with the exception of data collected for 1 mM acetyl-CoA which displays inhibition that is likely substrate inhibition. Due to limitations in experimental setup, we were unable to collect NgSAT rates for higher acetyl-CoA concentrations and as such do not feel comfortable fitting a substrate inhibition model due to only one point (1 mM acetyl-CoA) displaying inhibition. Enzyme concentration was factored into rates by dividing by the enzyme concentration to give the rate (s^{-1}). Plotted data points represent mean alongside SEM of two replicates.*

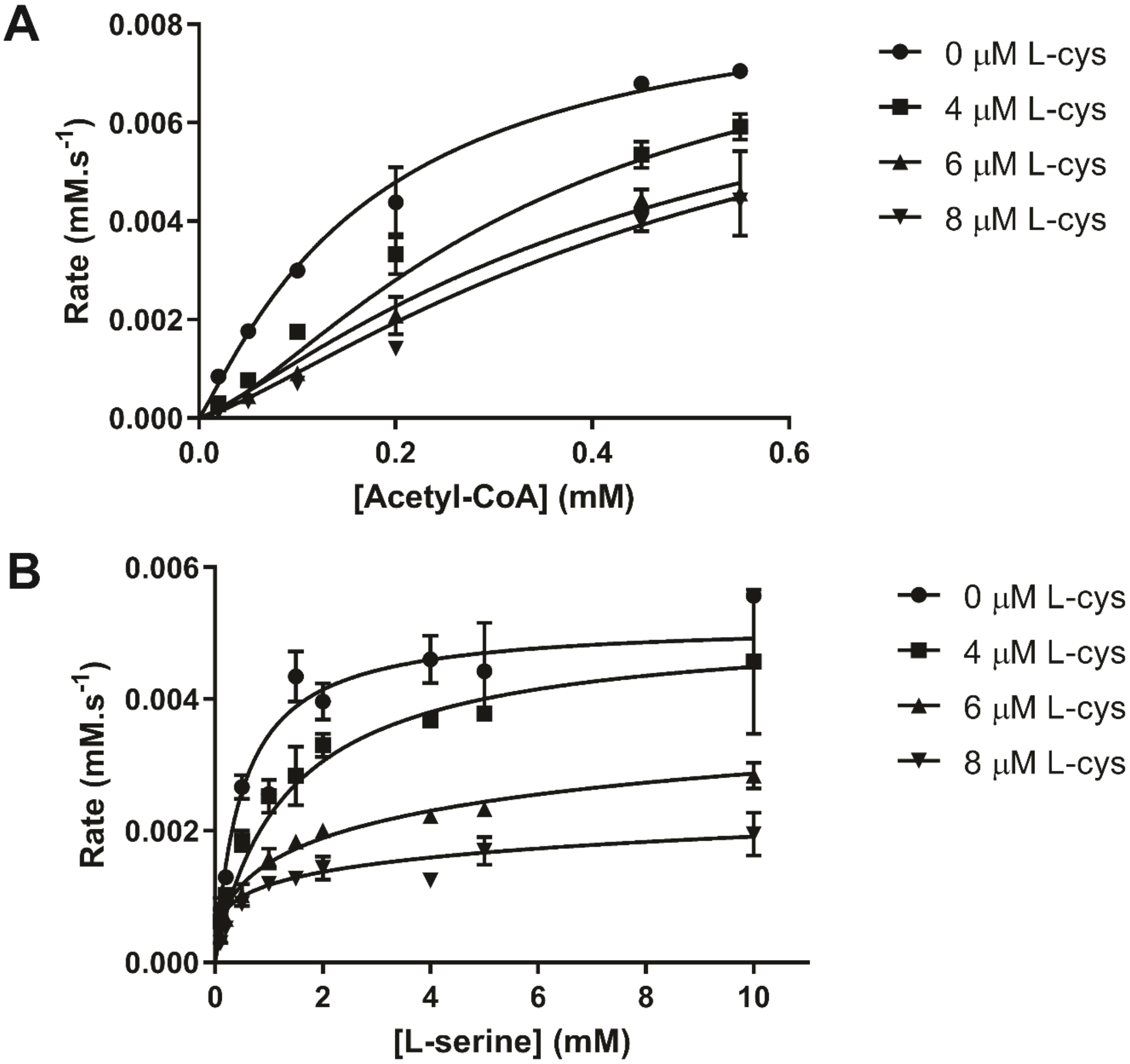

Supplementary Figure 5. *Characterization of cysteine inhibition, incorporation of the Hill coefficient to account for cooperativity. Data were collected for 0 (circles), 4 (squares), 6 (triangles) and 8 (inverted triangles) µM of L-cysteine. (A) Rates versus acetyl-CoA concentration plotted, with line representing competitive inhibition equation with incorporation of the hill coefficient. (B) Rates versus L-serine concentration plotted, with line representing competitive inhibition equation with incorporation of the hill coefficient. Data points are mean and error bars are SEM derived from two replicates.*

Supplementary Equation 1.

$$rate = \frac{V_{max} \cdot [S]^h}{[S]^h + {K_M}^h(1 + \frac{[I]}{K_i})}$$

[S] = substrate concentration

[I] = inhibitor concentration

h = hill coefficient

Supplementary Table 1. Competitive Inhibition + Hill coefficient values.

	**0 µM L-cys**	**4 µM L-cys**	**6 µM L-cys**	**8 µM L-cys**
**Acetyl-CoA**				
**h[a]**	1.137 ± 0.1507	1.405 ± 0.1162	1.203 ± 0.1127	1.275 ± 0.1080
**Ki[b] (µM)**	2.473 ±0.5823	2.473 ±0.5823	2.473 ±0.5823	2.473 ±0.5823
**R squared**	0.9851	0.9746	0.9791	0.9804
**L-serine**				
**h[a]**	1.005 ± 0.140	0.949 ± 0.081	0.481 ± 0.0553	0.298 ± 0.059
**Ki[b] (µM)**	2.508 ± 0.330	2.508 ± 0.330	2.508 ± 0.330	2.508 ± 0.330
**R squared**	0.9030	0.9096	0.9610	0.8035

[a] Hill coefficient < 1 indicates negatively cooperative binding ~ 1 indicates non-cooperative binding >1 indicates positively cooperative binding

[b] Inhibitory constant (Ki)

Error represents SEM of two replicates
